# Genome size, cytogenetic data and transferability of EST-SSRs markers in wild and cultivated species of the genus *Theobroma* L. (Byttnerioideae, Malvaceae)

**DOI:** 10.1371/journal.pone.0170799

**Published:** 2017-02-10

**Authors:** Rangeline Azevedo da Silva, Gustavo Souza, Lívia Santos Lima Lemos, Uilson Vanderlei Lopes, Nara Geórgia Ribeiro Braz Patrocínio, Rafael Moysés Alves, Lucília Helena Marcellino, Didier Clement, Fabienne Micheli, Karina Peres Gramacho

**Affiliations:** 1 Universidade Estadual de Santa Cruz, Ilhéus, Bahia, Brazil; 2 Cocoa Research Center, CEPLAC/CEPEC, Itabuna, Bahia, Brazil; 3 Universidade Federal de Pernambuco, Recife, Pernambuco, Brazil; 4 Embrapa Amazônia Oriental, Belém, Pará, Brazil; 5 Embrapa Recursos Genéticos e Biotecnologia, Distrito Federal, Brazil; 6 CIRAD, UMR AGAP, Montpellier, France; Huazhong University of Science and Technology, CHINA

## Abstract

The genus *Theobroma* comprises several trees species native to the Amazon. *Theobroma cacao* L. plays a key economic role mainly in the chocolate industry. Both cultivated and wild forms are described within the genus. Variations in genome size and chromosome number have been used for prediction purposes including the frequency of interspecific hybridization or inference about evolutionary relationships. In this study, the nuclear DNA content, karyotype and genetic diversity using functional microsatellites (EST-SSR) of seven *Theobroma* species were characterized. The nuclear content of DNA for all analyzed *Theobroma* species was 1C = ~ 0.46 pg. These species presented 2*n* = 20 with small chromosomes and only one pair of terminal heterochromatic bands positively stained (CMA^+^/DAPI^−^ bands). The small size of *Theobroma* ssp. genomes was equivalent to other Byttnerioideae species, suggesting that the basal lineage of Malvaceae have smaller genomes and that there was an expansion of 2C values in the more specialized family clades. A set of 20 EST-SSR primers were characterized for related species of *Theobroma*, in which 12 loci were polymorphic. The polymorphism information content (PIC) ranged from 0.23 to 0.65, indicating a high level of information per locus. Combined results of flow cytometry, cytogenetic data and EST-SSRs markers will contribute to better describe the species and infer about the evolutionary relationships among *Theobroma* species. In addition, the importance of a core collection for conservation purposes is highlighted.

## Introduction

The 22 species ascribed to the genus *Theobroma L*. (Malvaceae s.l.) are typically Neotropicals and distributed in the Amazon Tropical Forest. The genus *Theobroma* is monophyletic and a sister group of the *Herrania* genus, but the monophyly of *Theobroma* is weakly supported [1–3]. Molecular systematic studies suggested that the subfamily Sterculioideae (which traditionally included the genus *Theobroma*) is not monophyletic and it is divided into two clades: Byttnerioideae **(**Byttnerieae, Hermannieae, Lasiopetaleae and Theobromeae) and Sterculioideae *sensu strictu* (e.g., Dombeyeae, Sterculieae) [4, 5].

Nine species of *Theobroma* are present in the Brazilian Amazon, among them- cupuassu (*T*. *grandiflorum* Schum.) and cacao (*T*. *cacao* L.). The latter considered the most important species in the genus due to its economic value for providing raw material for production of chocolate and derivatives, cosmetics and pharmaceuticals [1, 6]. *T*. *grandiflorum*, one of the main tree crops in the Amazon region, has its pulp as the principal product, being used in juices, ice creams, yogurts and cosmetics. Their seeds can be used for *cupulate* production, an alternative to chocolate. [7, 8]. Recent studies also highlight the potential of cupuassu fruit extracts for medicinal use in gastrointestinal treatments [9].

A major limitation of *T*. *cacao* and *T*. *grandiflorum* production is witches’ broom disease, caused by *Moniliophthora perniciosa*, one of the most devastating diseases of cacao and cupuassu trees [10]. Several studies on the *T*. *cacao* L. vs. *M*. *perniciosa* interaction have been carried out to identify genes and proteins involved in mechanisms of fungus pathogenicity and/or plant resistance [11, 12]. Studies on wild *Theobroma* species might contribute to important discoveries regarding genes involved in resistance to various diseases, including witches’ broom, which may be useful for cacao breeding programs as well as functional guide for genome sequencing strategies, conservation programs or developing *ex situ* breeding designs [13, 14]. Functional microsatellites derived from expressed sequences tags, EST-SSR, can assist such selection. EST-SSRs have been used for traits characterization, breeding and mapping of quantitative trait loci (QTL's) [15–17]. Coming from coding regions, these markers are more conserved among populations of the same species and congeners, thus, enabling cross-amplification and allowing the characterization of molecular marker sets for species which have not been well characterized genetically [18–22]. Several EST-SSR markers have been identified for a diverse range of crops, such as maize [23] and tomato [24], arboreal crops as coffee [25, 26], cacao [27] and, the first work involving EST-SSR from *T*. *grandiflorum* [28]. Given their high levels of transferability between species [29, 30], the EST-SSR molecular markers are useful tool for studies of genetic diversity, functional genomics, and comparative mapping between species [31].

Cytogenetics based on chromosome variation and staining with chromomycin A3 (CMA) and 4’, 6-diamidino- 2-phenylindole (DAPI) [32] is a useful approach to study variations in plants. The fluorochrome CMA binds preferentially to GC-rich DNA sequences [33], whereas DAPI preferentially binds to AT-rich sequences [34]. Chromosome double staining with CMA^+^/DAPI^−^ has allowed the identification of AT- and GC-rich heterochromatin fractions in many plant groups [35]. Furthermore, genome size is a relevant information for understanding fundamental mechanisms and processes underlying plant growth, evolutionary, systematic taxonomic and cell biology studies, as well as detection of aneuploidy and apoptosis processes. In addition, it provides significant information to sequencing studies and characterization of novel molecular markers, such as EST-SSR [36–41].

Flow cytometry has been used to address questions related to differences in genome copy number. Previous studies quantified the 1C-Value (haploid DNA content) in four cacao genotypes at approximately 0.45 pg [42–44]. A more detailed comparative analysis of mitotic chromosomes of *T*. *cacao* and *T*. *grandiflorum* revealed small chromosomes (~2 μm), with only one pair of terminal heterochromatic bands, co-localized with the single 45S rDNA site and a single 5S rDNA site in the proximal region of the other chromosome pair [45]. These data suggest that the chromosomes of both species are conserved.

Therefore, the objectives of this research were to characterize seven *Theobroma* species using flow cytometry combined with cytogenetic and functional molecular markers from cacao. To our knowledge, this study is pioneer in the analysis of EST-SSRs marker transfer from cacao to the *Theobroma* species.

## Material and methods

### Biological samples

Leaves, seeds, and shoot apexes were obtained from cacao accessions at the Cacao Germplasm Bank (CGB) of the Cacao Research Center/Executive Commission of the Cacao Farming Plan—CEPEC/CEPLAC (Ilhéus, Bahia, Brazil). The *T*. *grandiflorum* genotypes were collected at Embrapa CPATU—Empresa Brasileira de Pesquisa Agropecuária (Belém, Pará, Brazil). Names of the accessions of the individuals used in this study are listed in Table 1.

**Table 1:** Accessions used for characterization, transferability of EST-SSR markers, genome size measurement and cytogenetics analyses.

Species	Accession	Collection sites	Place of origin	Analysis
*T*. *cacao*	P4B	CGB CEPLAC/CEPEC-Ilhéus, Bahia	Peru	EST-SSR
*T*. *cacao*	Na33	CGB CEPLAC/CEPEC-Ilhéus, Bahia	Peru	EST-SSR
*T*. *cacao*	CCN51	CGB CEPLAC/CEPEC-Ilhéus, Bahia	Ecuador	EST-SSR
*T*. *cacao*	Ma16	CGB CEPLAC/CEPEC-Ilhéus, Bahia	Brazil	EST-SSR
*T*. *cacao*	MOQ216	CGB CEPLAC/CEPEC-Ilhéus, Bahia	Ecuador	EST-SSR
*T*. *cacao*	CSul3	CGB CEPLAC/CEPEC-Ilhéus, Bahia	Brazil	EST-SSR
*T*. *cacao*	OC67	CGB CEPLAC/CEPEC-Ilhéus, Bahia	Venezuela	Cytometry/ EST-SSR
*T*. *cacao*	EET399	CGB CEPLAC/CEPEC-Ilhéus, Bahia	Ecuador	EST-SSR
*T*. *cacao*	TSH1188	CGB CEPLAC/CEPEC-Ilhéus, Bahia	Trinidad/Tobago	EST-SSR
*T*. *cacao*	ICS1	CGB CEPLAC/CEPEC-Ilhéus, Bahia	Trinidad/Tobago	Cytometry/ EST-SSR
*T*. *cacao*	SPA12	CGB CEPLAC/CEPEC-Ilhéus, Bahia	Colombia	EST-SSR
*T*. *cacao*	RB36	CGB CEPLAC/CEPEC-Ilhéus, Bahia	Brazil	EST-SSR
*T*. *cacao*	SCA6	CGB CEPLAC/CEPEC-Ilhéus, Bahia	Peru	Cytometry/ EST-SSR
*T*. *cacao*	Catongo	CGB CEPLAC/CEPEC-Ilhéus, Bahia	Brazil	EST-SSR
*T*. *cacao*	GU171	CGB CEPLAC/CEPEC-Ilhéus, Bahia	French Guiana	EST-SSR
*T*. *cacao*	Rosa Maria	CGB CEPLAC/CEPEC-Ilhéus, Bahia	*	EST-SSR
*T*. *cacao*	MO20	CGB CEPLAC/CEPEC-Ilhéus, Bahia	Peru	EST-SSR
*T*. *cacao*	UF 20	CGB CEPLAC/CEPEC-Ilhéus, Bahia	Costa Rica	Cytometry
*T*. *cacao*	UF 667	CGB CEPLAC/CEPEC-Ilhéus, Bahia	Costa Rica	Cytometry
*T*. *cacao*	RIM 24	CGB CEPLAC/CEPEC-Ilhéus, Bahia	México	Cytometry
*T*. *cacao*	Pentagona	CGB CEPLAC/CEPEC-Ilhéus, Bahia	*	Cytometry
*T*. *cacao*	OC 61	CGB CEPLAC/CEPEC-Ilhéus, Bahia	Venezuela	Cytometry
*T*. *cacao*	ICS 100	CGB CEPLAC/CEPEC-Ilhéus, Bahia	Trinidad/Tobago	Cytometry
*T*. *cacao*	CEPEC 515	CGB CEPLAC/CEPEC-Ilhéus, Bahia	Brazil	Cytometry
*T*. *grandiflorum*	C174	EMBRAPA-CPATU-Belém, Pará	Brazil	EST-SSR
*T*. *grandiflorum*	C1074	EMBRAPA-CPATU-Belém, Pará	Brazil	EST-SSR
*T*. *grandiflorum*	*T*. *grandiflorum*	CEPLAC/CEPEC-Ilhéus, Bahia	Brazil	Cytometry
*T*. *bicolor*	*T*. *bicolor*	CEPLAC/CEPEC-Ilhéus, Bahia	Brazil	Cytometry/ EST-SSR
*T*. *microcarpum*	*T*. *microcarpum*	CEPLAC/CEPEC-Ilhéus, Bahia	Brazil	Cytometry/ EST-SSR
*T*. *obovatum*	*T*. *obovatum*	CEPLAC/CEPEC-Ilhéus, Bahia	Brazil	Cytometry/ EST-SSR
*T*. *speciosum*	*T*. *speciosum*	CEPLAC/CEPEC-Ilhéus, Bahia	Brazil	Cytometry/ EST-SSR
*T*. *subincanum*	*T*. *subincanum*	CEPLAC/CEPEC-Ilhéus, Bahia	Brazil	Cytometry/ EST-SSR

*Unknown information

### Chromosome banding

Root tips obtained from seeds or apical meristems were pre-treated with 0.05% colchicine during 24 h at 10°C and fixed in ethanol:acetic acid (3:1; v/v) for 2–24 h at room temperature and then stored at -20°C. Afterward, the fixed root tips were washed in distilled water and digested in a 2% (w/v) cellulase (Onozuka)/20% (v/v) pectinase (Sigma) solution at 37°C for 90 min. The apical shoots were macerated in a drop of 45% acetic acid and the coverslip removed in liquid nitrogen. For CMA^+^/DAPI^−^ bands double staining, the slides were aged for three days, stained with 10 μL of CMA 0.1 mg/mL for 30 min, and restained with 10 μL of DAPI 2g/mL for 60 min [46]. The slides were mounted in glycerol: McIlvaine buffer pH 7.0 (1:1) and aged for three days before analysis in an epifluorescence Leica DMLB microscope. The images were captured with a Cohu CCD video camera using the Leica QFISH software and later edited in Adobe Photoshop CS3 version 10.0.

### Flow cytometry

A suspension of nuclei from young leaves was prepared as described by [47] using Tris-MgCl_2_ (WPB buffer). The genome size was estimated using a CyFlow SL flow cytometer (Partec, Görlitz, Germany). For the determination of DNA content, proportionality to fluorescence intensity was assumed and calculated based on at least three different measurements for each individual sample. The histograms were generated in the FloMax (Partec) software using the fluorescence pulse area histogram for analysis. The G1 peak of a diploid *S*. *lycopersicum “*Stupicke” (1.96 pg DNA content), was used as standard sample, and, set to channel 50 in the 1000 channel histogram. The *S*. *lycopersicum* seeds were obtained from the Institute of Experimental Botany, Olomouc, Czech Republic [48]. The genome size in base pair for each species and the 1C value of the genome was estimated based on the genome size of *S*. *lycopersicum*. In order to compare the genome sizes of the different species, a covariance analysis was performed using the GLM procedure of the SAS software (SAS Institute Inc., 2004). The genome size of *S*. *lycopersicum* was used as covariate to improve the accuracy of the analysis [49].

### DNA extraction

The DNA extraction was performed using healthy leaves in an intermediary stage of maturation with the MATAB protocol [50] slightly modified for *Theobroma* species other than cacao. Approximately, 300 mg of leaves were ground using metal beads [51] in the presence of liquid nitrogen. Then, 800 μL of extraction buffer (1.4 M NaCl, 100 mM Tris-HCl pH 8.0, 20 mM EDTA, 10 mM Na_2_SO_3_, 1% PEG 6000, 2% MATAB) preheated at 74°C were added to the macerate, and incubated for 1 h at 65°C. After, the sample was cooled at room temperature and 800 μL of chloroform:isoamyl alcohol (24:1, v/v) was added to each sample. The samples were then centrifuged at room temperature for 10 min at 14.000 rpm and the supernatants collected. Afterwards, 700 μL of cold isopropanol was added to the samples and centrifuged for 10 min at 14000 rpm. The pellets were collected and transferred to news 2 mL tubes containing 100 μL of TE (10 mM Tris-HCl, pH 8.0, 1 mM EDTA) in 40 μg/mL RNAse, The integrity of the DNA samples was checked on 1% agarose gel stained with 1 ng/μL of gel red. Quantification of the DNA samples was performed using the Picodrop Microliter UV/Vis Spectrophotometer (Picodrop Limited, UK). The samples had their concentration adjusted to 1 ng DNA/μL and stored at -20°C.

### Microsatellite loci, amplification by PCR and genotyping

A total of 55 EST-SSR primers previously developed specifically for cacao [15–16, 27] in our laboratory, were synthesized and used to test transferability to cupuassu genotypes. From these, a total of 20 EST-SSR were selected according to the following criteria: a minimum size of 14 bp or originating from genes or proteins involved in mechanisms of plant resistance, or both. Seventeen *T*. *cacao* accessions representing the diversity of the main genetic *T*. *cacao* groups were analyzed [17, 52], two *T*. *grandiflorum* genotypes and five genotypes of wild *Theobroma* (Table 1). These accessions belong to CEPLAC genetic breeding program [17, 21]. After validation on cacao, the markers were tested for transferability on *T*. *grandiflorum* genotypes 174 (Coari) and 1074, resistant and susceptible to witches’ broom disease, respectively. These genotypes are genitors of several progenies in cupuassu breeding program [28] (Table 1).

PCR reactions were carried out with 30 ng of DNA, 0.2 mmol/L of each primer, 2.0 mmol/L of MgCl_2_, 0.2 mmol/L of each dNTP, 1X buffer and one unit of DNA Taq polymerase (Ludwig Biotecnologia Ltda) in a final volume of 20 μL. PCRs were carried out using touchdown protocol with 10 cycles as follow: denaturation at 94°C for 4 min, annealing temperature at 60–48°C /56°C with 1°C decrease at each cycle, and extension at 72°C for 1 min and 30 s, followed by 30 cycles at 94°C for 30 s, 48°C for one min and 30 s, and final extension of 4 min at 72°C. Polymorphism evaluation was carried out by electrophoresis on 6% denaturing TBE acrylamide gel using as running buffer TBE 1X (89 mmol/L Tris, mmol/L boric acid and 2 mmol/L of EDTA). Microsatellite polymorphism was visualized using the silver staining method [53, 54].

The amplified SSR DNA bands representing different alleles were scored on different genotypes. A principal component analysis, conducted on the allele frequency data, number of allele per locus (Na) and average observed heterozygosity (Ho) were determined with the GENETIX software v. 4.05.2 [55]. The polymorphic information content (PIC) was obtained for each locus with the CERVUS software version 3.0 [56, 57], and the genetic distance (D) was calculated according to Nei Genetic Distances [58]. The genetic analyses were carried out considering three groups: *T*. *cacao*, *T*. *grandiflorum* and *Theobroma* wild species.

## Results

### Chromosome number, chromosome banding and DNA content

**Fig 1:**
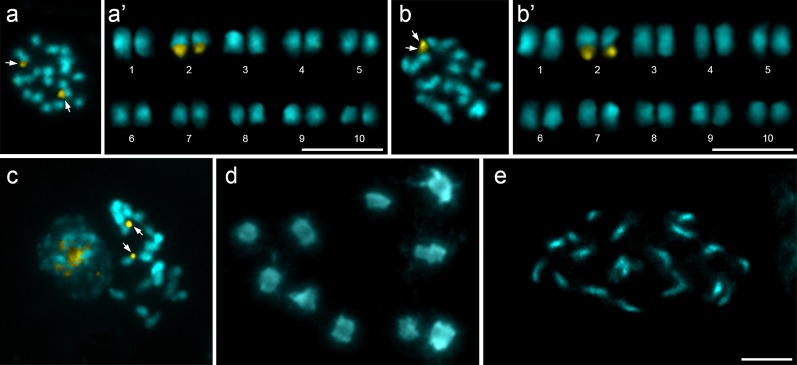
Cytogenetic analysis of the genus *Theobroma*. (a-a’) metaphase and caryogram of the *T*. *cacao* ssp leicocarpum stained with CMA (yellow) and DAPI^−^ (blue). (b-b’) Metaphase and caryogram of *T*. *cacao* cv. Scavina 6. (c) metaphase of *T*. *bicolor* stained with CMA^+^/DAPI^−^. (d) diakinesis of *T*. *bicolor* showing the 10 pair sofbivalent. (e) prometaphase of *T*. *grandiflorum*, bars = 5 μm. Arrows in a, b, c points to the CMA^+^ bands.

All species showed symmetric karyotypes 2n = 20 with small metacentric/submetacentric chromosomes. In all species, a CMA^+^/DAPI^−^ band was present on the terminal region of the long arm of a single chromosome pair. This CMA^+^ band was frequently heteromorphic in size and distended in one or both homologues (Fig 1).

**Fig 2:**
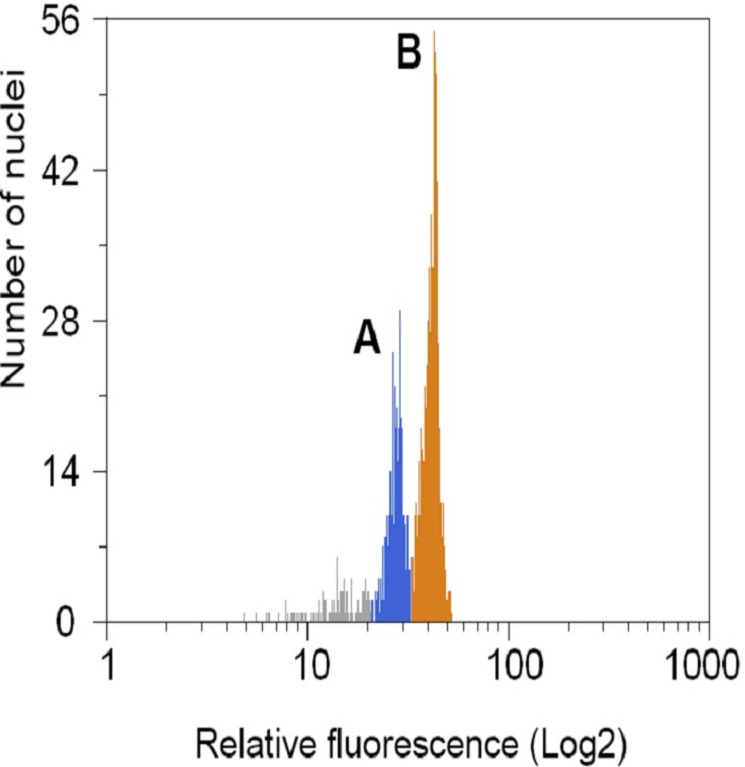
Histogram of relative nuclear DNA content (genome size). (A) Nuclear DNA content in *T*. *cacao* cv. ICS100. (B) *S*. *lycopersicum* cells, included as an internal standard. Nuclei isolated from cocoa leaves were stained with propidium iodide and analyzed by flow cytometry.

The 1C nuclear DNA content and genome size of the plant species are presented in Table 2, and the histogram of the fluorescence peak are presented in Fig 2.

**Table 2:** Genome size of *Theobroma* species.

Species	Genome size (1C value)	Number of base pairs
*T*. *cacao*	0.4666	456 Mpb
*T*. *grandiflorum*	0.4589	448 Mpb
*T*. *microcarpum*	0.4624	452 Mpb
*T*. *obovatum*	0.4624	452 Mpb
*T*. *speciosum*	0.4624	452 Mpb
*T*. *bicolor*	0.4624	452 Mpb
*T*. *subincanum*	0.4624	452 Mpb
**Average**	**0.46**	

Nuclear DNA content (1C values) in the studied *Theobroma* species ranged from 0.41 pg in *T*. *microcarpum* to 0.49 pg in *T*. *speciosum* and *T*. *subincanum*, with an average of 0.46 pg (Table 2). Three species (*T*. *bico*lo*r*, *T*. *obova*tum and *T*. *grandiflorum*) showed 1C = 0.48. The ten *T*. *cacao* genotypes analyzed showed 1C values ranging from 0.44 to 0.47 pg, with an average of 0.45 pg. The coefficient of variation (CV) was low (4.07%), and the estimated genome sizes of the seven *Theobroma* species, after adjustment by the genome size of *S*. *lycopersicum* were not statistically different by the F test (p-value = 0.975) (S1 Table).

### Cross amplification and genetic analyses

The 20 EST-SSR markers produced informative results about allelic variation among the cultivated and wild species. Twelve of them (60%) were polymorphic and 8 monomorphic (40%) in *T*. *cacao* individuals. The 20 primer pairs tested showed satisfactory cross-amplification results in 75% of *T*. *grandiflorum and T*. *subincanum*, 90% in *T*. *obovatum*, 60% in *T*. *bicolor and* 35% in *T*. *speciosum* and *T*. *microcarpum*. The annealing temperature of the primer pairs ranged from 48–60°C with amplicons varying from 100 to 322 pb (S1 Table).

Considering all 24 sampled individuals and the 20 loci, 16 were polymorphic and 4 were monomorphic. The analysis using the 16 polymorphic EST-SSRs revealed a total of 24 alleles, with an amplitude of size ranging from 155–165 (mEstTcCepec13-3) to 250–260 base pairs (msEstTsh-10) (S2 Table). The number of alleles per locus ranged from two (mEstTcCepec60, mEstTcCepec16-8, mEstTcCepec24, mEstTcCepec13, mEstTcCepec31, mEstTcCepec20-5) to seven (mEstTcCepec16-4), and the polymorphic information content (PIC) of each locus, excluding the monomorphic ones, ranged from 0.08 (mEstTcCepec31) to 0.75 (mEstTcCepec13-4). The loci were classified either as moderately polymorphic with 0.25 > PIC > 0.5 or highly polymorphic with PIC > 0.5 (S2 Table) [53]. The average number of alleles per locus varied among the *Theobroma* groups, ranging from 2.4 in *T*. *cacao* to 1.8 in *Theobroma* ssp and 1.1 in *T*. *grandiflorum* (Table 3).

**Table 3:** Genetic diversity estimates for individuals of the *Theobroma* genus.

Groups	Na	He	H_O_
*T*. *cacao*	2.4	0.31	0.12
*T*. *grandiflorum*	1.1	0.04	0.00
T. ssp	1.8	0.28	0.06
**Average**	**1.8**	**0.21**	**0.06**

Na = number of alleles averaged over all loci; H_O_ = unbiased observed heterozygosity (Nei, 1978); He = unbiased expected heterozygosity (Nei, 1978).

The genetic distance also varied among the *Theobroma* groups. *Theobroma cacao* group was more genetically distant from the *T*. spp group (0.444) and less distant from the *T*. *grandiflorum* group (0.219). *Theobroma grandiflorum* and *T*. spp groups had a moderate genetic distance of 0.378 (Table 4).

**Table 4:** Genetic distance matrix between the three groups of individuals of the genus *Theobroma*.

Groups	*T*. *cacao*	*T*. *grandiflorum*	*T*. ssp
*T*. *cacao*	0.000	--	--
*T*. *grandiflorum*	0.219	0.000	--
*T*. ssp	0.444	0.378	0.000

## Discussion

### Genome size and cytogenetics

In general, the genome size among related species is, for the most part, stable and rarely exceeds values above or below 2 to 5% when using techniques commonly suggested for determining nuclear DNA content [59]. This trend was also observed in this study, wherein the genome sizes estimated in this study for species of the *Theobroma* genus (*T*. *cac*ao, *T*. *grandiflorum*, *T*. *microcarpum*, *T*. *speciosum*, *T*. *bicolor*, *T*. *subincanum* and *T*. *obovatum)* did not differ statistically. For most of these species, there are no previous studies about the genome size, except for *T*. *cacao*. The small differences found in the genome sizes in samples of *T*. *cacao* have been reported in previous studies [42, 43, 60]. Nevertheless, these differences in measurements of DNA content observed between laboratories cannot be interpreted as interspecific variation, because the measuring tools may present small differences in the alignment along the time. Regarding the variations among species, small differences in measurements can be generated by phenolic compounds, which have antioxidant activity, thus conferring protection against DNA damage to the cells. These compounds are known to inhibit proper DNA staining. In experiments that aim measuring the size of the genome, any failure in the application of the protocol may cause variations in the data obtained, in comparison to homogeneous values [61–64]. However, in this study it was possible to acquire a high resolution of the histograms. Besides replications of samples, other actions were implemented, like use of *S*. *lycopersicum* genome as control to increase the accuracy of the measurements [65–68]. Besides that, the coefficient of variation was low (4.07%), suggesting a high precision in the comparisons. Therefore, in the present case, the homogeneity of the genome sizes of *Theobroma* species suggests a true uniformity in those sizes.

Variations in genome size of angiosperms are wide, varying from 1C = 0.06 pg in *Genlisea margaretae* to 1C = 152.23 pg in *Paris japonica*, with an extensive variation occurring within the genus [68, 69]. Some studies have revealed that chromosome length ranged from 2.00 to 1.19 μm for *T*. *cacao* and from 2.21 to 1.15 μm for *T*. *grandiflorum*, with most chromosome pairs similar in morphology and size, corroborating with our findings [45]. Plant species native from tropical forests tend to have a reduced genome size, which could be influenced by differences in temperature affecting cellular division and expansion [70]. The process of cellular division and growth are favored by elevated temperatures as seen in cacao and other species of the genus [71]. This process can select a more short mitotic cycle and consequently small cells and small genomes compared to plants of temperate regions [42].

Studies show that probably the ancestral genome size in Angiosperms was reduced, and that along the evolutionary scale DNA content suffered increases due to polyploidization events and self-replicating DNA elements. The reduced genome sizes observed in the studied species may have also a phylogenetic signal. The clade Byttnerioideae (which includes *Theobroma* and *Herrania*) presents small genomes (~0.43 pg), as well as it is related to the Grewioideae (~0.48 pg) clade [72–74]. Byttnerioideae and Grewioideae form the most basal group of Malvaceae, suggesting that the small genomes are a plesiomorphyc condition in the family. In contrast, the most specialized clades within Malvaceae, Sterculioideae (~1.944 pg), Bombacoideae (1.97 pg) and Malvoideae (1.72 pg) have much larger genomes, suggesting that there was a trend of expansion of the genome size in these lineages [3]. In some cases, this increase in the genome can be influenced by polyploidy events, such as for Bombacoideae [1, 75].

The small genomes in these species studied here may be correlated with the karyotype stability in the genus. Our data support the karyotypes already described for other species in the genus, that it is a taxon predominantly diploid (2n = 20), with small and symmetric chromosomes [44, 76]. The only differences reported in the literature are detectable cytological heterochromatic bands observed on the centromeric/ pericentromeric regions of all 20 chromosomes of cacao after C-banding stained with either Giemsa or DAPI, whereas never being detected in *T*. *grandiflorum*. The presence of only a couple of bands CMA^+^/DAPI^−^in all species, and in different varieties of cacao also confirms the karyotype correlations among them and are in agreement with previous analyses [45].

### Characterization and transferability of EST-SSR markers

Adoption of genomic approaches to crop improvement studies and preservation of wild tree species is severely limited by the lack of sufficient molecular markers. EST-SSRs are codominant, highly reproducible and polymorphic markers. With these characteristics, they have been used favorably for population genetic analyses and genetic mapping in several species.

Rate of polymorphism in genomic SSRs is generally high in comparison to SSR from ESTs [77, 78]. However, EST-SSR shows some advantages, such as higher frequency in the genome and their link to interesting agronomical traits [79]. From the 20 EST-SSR tested here, 60% generated polymorphic loci. In previous studies lower rates were found, when 32 EST-SSRs associated with resistance to *M*. *perniciosa* were tested only 26.7% were polymorphic, nevertheless the individuals used in the present study were more heterogeneous [15].

Regarding the genetic diversity found in the sampled individuals, we noted that the highest polymorphic information content (PIC) was in the *T*. *cacao* group, showing the potential coverage of these markers. It is worth mentioning that it was not the aim of this study to characterize the genetic structure of populations in the *Theobroma* genus, but to test the cross-amplification to wild species of *Theobroma* genus and to *T*. *grandiflorum*. One of the advantages of EST-SSR markers is the fact they are easily transferable between species of the same genus, since they are derived from commonly conserved transcribed regions of DNA, directly decreasing in the costs of molecular studies in wild species [80–82]. In the present work, the transferability rate of theses EST-SSRs markers among the *Theobroma* species ranged from 35% to 90% among samples. Therefore, based on the premise that EST-SSRs are highly conserved in congeners, the percent of functional primers to other species of the genus decreased with the increase in genetic distance, that is, *T*. *microcarpum* from the section Telmatocarpus and *T*. *speciosum* from the section Oreanthes showed the lowest rates of primer transferability (35%). In a work with wheat, it was reported that this is an expected result due to the insertion of introns in correlated species [83] that modify the target sequence, however, with rates of amplification normally higher than 50% [31].

Microsatellite markers are especially useful to characterize the genetic diversity and kinship between species, due the high polymorphism and number of alleles per locus [84]. *Theobroma grandiflorum* and *T*. *obovatum* species belong to section Glossopetalum, considered the most ancestral section of the genus, while *T*. *cacao* (section *Theobroma*) suggests a distant evolutionary relationship between these species. Nevertheless, there have been reports of hybrids between *T*. *cacao* and *T*. *grandiflorum* and *T*. *grandiflorum* with *T*. *obovatum* meaning closer relationship between those species [3, 85].

The first sets of molecular markers reported for wild species of *Theobroma* were RAPD (Random Amplified Polymorphic DNA) used to elucidate phylogenetic relationships between species. Patterns of RAPD showed intra and interspecific polymorphism and some bands of similar lengths between species classified in the same section or correspondents [86].

Previous studies found a transferability rate of SRR markers from *T*. *cacao* to *T*. *grandiflorum* of 60.4%, showing similarity between species and highlighting the possibility of using those markers in association mapping and breeding [87]. In the present study, the transferability rate of EST-SSRs from *T*. *cacao* to *T*. *grandiflorum* was slightly higher, around 70%, as expected, considering that EST-SSRs come from conserved sequences. Regarding *T*. *obovatum*, the rate of cross-amplification was 90%. Until now, there is no previous report of SSR or EST-SSR markers to this species. These cross-amplification rates are in agreement with previous studies that have shown that the proportion of cross-amplification studies involving polymorphic SSR loci within the same plant genus ranged from 20% to 100% [88].

The constancy of the genome size of theses *Theobroma* species and the high rate of EST-SSR cross-amplification supports our conclusion that the areas bordering the microsatellites in the studied species are conserved enough to allow cross-amplification. Additionally, suggesting that these species have been conserved along the evolution regarding their genomic sequences and number and size of chromosomes, which are relatively small compared to most angiosperms. This information can also be confirmed by the amount of non-coding repetitive DNA, composed of transposable elements, satellite DNA, introns and pseudo genes, as seen for the *T*. *cacao* genome [89–91]. Preservation of the sequence depends mainly on the evolutionary relationship between the species of origin. Thus, the more diverse the taxon, the less successful the cross-amplification will be. Data from this research allows inferences that these *Theobroma* species have a certain genomic homology.

The EST-SSR markers evaluated in this study showed a considerable transferability rate into six related *Theobroma* species, therefore, these markers are important to closely monitor the genetic variability. In fact, this is the first report of EST-SSR molecular markers used in wild species of the *Theobroma* genus, thus, representing a new set of primers highly informative that can be used in studies of transferability, paternity, and genetic flow of Amazon species. They can also help in studies aiming better strategies of biodiversity conservation and studies of disease resistance genes in *T*. *grandiflorum* [92].

## Supporting information

S1 Table Analysis of covariance for the genome size measured in seven species of *Theobroma*. (DOCX)

S2 Table Characteristics of 20 EST-SSRs markers used on the present work. Shown for each primer pair are the previously published forward and reverse sequence, repeat type, allelic amplitude (bp), number of alleles (Na) and polymorphic information content (PIC) for species of *Theobroma*.
(DOC)
