# Supplementary material for: Genome size, cytogenetic data and transferability of EST-SSRs markers in wild and cultivated species of the genus Theobroma L. (Byttnerioideae, Malvaceae)
Source: PLoS One. 2017 Feb 10;12(2):e0170799. doi: 10.1371/journal.pone.0170799 (PMC5302445; doi:10.1371/journal.pone.0170799)
Supplement: S1 Table — (DOCX) [file pone.0170799.s001.docx]

**S1 Table. Analysis of covariance for the genome size measured in seven species of *Theobroma***

| **Source** | **DF** | **SS** | **MS** | **F** | **P –value** |
| --- | --- | --- | --- | --- | --- |
| *Theobroma* species | 6 | 0.0017 | 0.0004 | 0.2000 | 0.9746 |
| Standard sample | 1 | 0.0004 | 0.0313 | 0.2600 | 0.6148 |
| Error | 22 | 0.0313 | 0.0014 |  |  |
| Total | 29 | 0.0806 |  |  |  |
