# Supplementary material for: Genome size, cytogenetic data and transferability of EST-SSRs markers in wild and cultivated species of the genus Theobroma L. (Byttnerioideae, Malvaceae)
Source: PLoS One. 2017 Feb 10;12(2):e0170799. doi: 10.1371/journal.pone.0170799 (PMC5302445; doi:10.1371/journal.pone.0170799)
Supplement: S2 Table — Shown for each primer pair are the previously published forward and reverse sequence, repeat type, allelic amplitude (bp), number of alleles (Na) and polymorphic information content (PIC) for species of Theobroma. (DOC) [file pone.0170799.s002.doc]

***S2 Table. Characteristics of 20 EST-SSRs markers used on the present work. Shown for each primer pair are the previously published forward and reverse sequence, repeat type, allelic amplitude (bp), number of alleles (Na) and polymorphic information content (PIC) for species of Theobroma.***

| **Locus** | **Sequence (5'-3')** | | **Motif** | | **Allelic Amplitude (bp)** | | | ***T. cacao***  **(17)** | | | | ***T. grandiflorum* (2)** | | | | | ***T*. ssp**  **(5)** | | | | | | **Total (24)** | | | | | | **Reference** | | |  |
| --- | --- | --- | --- | --- | --- | --- | --- | --- | --- | --- | --- | --- | --- | --- | --- | --- | --- | --- | --- | --- | --- | --- | --- | --- | --- | --- | --- | --- | --- | --- | --- | --- |
|  |  | |  | |  | | | **Na** | | **PIC** | | **Na** | | **PIC** | | | **Na** | | | **PIC** | | | **Na** | | | **PIC** | | |  | | |  |
| mEstTcCepec16-4 | | F: TCAATTCCAACACCAAAAC | | [AG]4N24[AGA]5  [AAG]4 | | | 200-213 | 7 | | 0.67 | | 1 | | 0 | | 1 | | | 0 | | | 7 | | | 0.55 | | | [15]* | | |  | |
|  | | R: CTGATCTGGGTCTTTGTTCA | |  | | |  |  | |  | |  | |  | |  | | |  | | |  | | |  | | |  | | |  | |
| mEstTcCepecR20-4 | | F: TCAGAACATTTGCACATCA | | [GA]4N42[TA]4 | | | 204-210 | 1 | | 0 | | 1 | | 0 | | 2 | | | 0.23 | | | 2 | | | 0.05 | | | [15]* | | |  | |
|  | | R: TACAGTTACCCCAAGGATGA | |  | | |  |  | |  | |  | |  | |  | | |  | | |  | | |  | | |  | | |  | |
| msEstTsh-10 | | F: ACCCCTCAATCTCACACATA | | [CT]10 | | | 250-260 | 3 | | 0.59 | | 0 | | 0 | | 3 | | | 0.55 | | | 5 | | | 0.7 | | | [16] | | |  | |
|  | | R: GCTTGGCGCTCTTAGTATC | |  | | |  |  | |  | |  | |  | |  | | |  | | |  | | |  | | |  | | |  | |
| mEstTcCepec47 | | F: CTATGATTTCACTCCCCAAC | | [CT]10 | | | 220-238 | 4 | | 0.53 | | 0 | | 0 | | 4 | | | 0.6 | | | 5 | | | 0.58 | | | In this study* | | |  | |
|  | | R: ATCATAGCCTTATCGCATTC | |  | | |  |  | |  | |  | |  | |  | | |  | | |  | | |  | | |  | | |  | |
| mEstTcCepec60 | | F: GGAAGGGCTTAGTTTTAGAG | | [CT]4N30[GAG]5 | | | 163-175 | 2 | | 0.37 | | 0 | | 0 | | 3 | | | 0.59 | | | 4 | | | 0.5 | | | In this study* | | |  | |
|  | | R: GGATCGGAGAGTATTAGGAT | |  | | |  |  | |  | |  | |  | |  | | |  | | |  | | |  | | |  | | |  | |
| mEstTcCepec16-8 | | F: TTCTTGTCCTTCCCTCTCTC | | [CT]4N40[TC]4 | | | 210-230 | 2 | | 0.12 | | 1 | | 0 | | 1 | | | 0 | | | 2 | | | 0.33 | | | [15]* | | |  | |
|  | | R: CCAGTCAAAACACCTAACCA | |  | | |  |  | |  | |  | |  | |  | | |  | | |  | | |  | | |  | | |  | |
| mEstTcCepec13-1 | | F: ACTGATGAGAGGGTAGCTGT | | [CTTT]9 | | | 100-190 | 1 | | 0 | | 1 | | 0 | | 2 | | | 0.3 | | | 2 | | | 0.11 | | | [15]* | | |  | |
|  | | R: AGTCCTCCTTTTCCTTCAAA | |  | | |  |  | |  | |  | |  | |  | | |  | | |  | | |  | | |  | | |  | |
| mEstTcCepec24 | | F: GATTTCTTTTCTTCGCTTCC | | [CT]4 | | | 320-322 | 2 | | 0.37 | | 1 | | 0 | | 1 | | | 0 | | | 2 | | | 0.37 | | | [27] | | |  | |
|  | | R: AGACTGGGTTTTAGCTCCAC | |  | | |  |  | |  | |  | |  | |  | | |  | | |  | | |  | | |  | | |  | |
| mEstTcCepec13 | | F: TGCTTAAGGAGGTGTTTGAC | | [AG]4 | | | 200-204 | 3 | | 0.49 | | 2 | | 0.3 | | 2 | | | 0.37 | | | 3 | | | 0.52 | | | [27] | | |  | |
|  | | R: GAATCACCCTCCTTGATTTC | |  | |  | | |  | |  | |  | |  | | |  | | |  | | |  | | |  | | |  | | |

**S2 Table (continuation...). Characteristics of 20 EST-SSRs markers used on the present work. Shown for each primer pair are the previously published forward and reverse sequence, repeat type, allelic amplitude (bp), number of alleles (Na) and polymorphic information content (PIC) for species of *Theobroma.***

| **Loci** | **Sequence (5'-3')** | | **Motif** | | **Allelic Amplitude (bp)** | | ***T. cacao***  **(17)** | | ***T. grandiflorum* (2)** | | ***T*. ssp**  **(5)** | | **Total (24)** | | **Reference** | |
| --- | --- | --- | --- | --- | --- | --- | --- | --- | --- | --- | --- | --- | --- | --- | --- | --- |
|  |  | |  | |  | | **Na** | **PIC** | **Na** | **PIC** | **Na** | **PIC** | **Na** | **PIC** |  | |
| mEstTcCepec13-4 | | F: AAGCACAACCAAAGACAAAA | | [CTG]6 | | 157-166 | 3 | 0.45 | 1 | 0 | 4 | 0.67 | 4 | 0.75 | [15]* |  |
|  | | R: ACTTTGGGTGGAAAATGAAT | |  | |  |  |  |  |  |  |  |  |  |  |  |
| mEstTcCepec13-5 | | F: GGGGAAAGAAGTTGGTTTTA | | [GA]4N18[AG]4 | | 208-210 | 1 | 0 | 1 | 0 | 1 | 0 | 2 | 0.22 | [15]* |  |
|  | | R: CTCAAATCTCTCCCTCCCTA | |  | |  |  |  |  |  |  |  |  |  |  |  |
| mEstTcCepec31 | | F: ACTGATGAGAGGGTAGCTGT | | [CTG]4 [CTG]7 | | 194-200 | 2 | 0.06 | 1 | 0 | 2 | 0.16 | 3 | 0.08 | [27] |  |
|  | | R: AGTCCTCCTTTTCCTTCAAA | |  | |  |  |  |  |  |  |  |  |  |  |  |
| mEstTcCepec20-5 | | F: AGAAAATGTTCCATCCACAA | | [AG]7 | | 245-249 | 3 | 0.41 | 1 | 0 | 2 | 0.26 | 3 | 0.5 | [15]* |  |
|  | | R: GGAGAGGAAAGGCTACTTCA | |  | |  |  |  |  |  |  |  |  |  |  |  |
| mEstTcCepec1 | | F: CAGGCCTTTATTTGTCACAC | | [CT]4 | | 158-166 | 4 | 0.53 | 1 | 0 | 1 | 0 | 5 | 0.61 | [27] |  |
|  | | R: TATTGTCGTCGCTGATACCT | |  | |  |  |  |  |  |  |  |  |  |  |  |
| mEstTcCepec13-3 | | F: AATTCAAGCCCAAATCTACC | | [CAG]10 | | 155-165 | 5 | 0.56 | 0 | 0 | 0 | 0 | 5 | 0.56 | [15]* |  |
|  | | R: TGGAGACATGTTTCATAGGG | |  | |  |  |  |  |  |  |  |  |  |  |  |
| mEstTcCepec2 | | F: AGGGGTGTTTTTATTGTCGT | | [CT]4[CT]4 | | 210 | 1 | 0 | 1 | 0 | 1 | 0 | 1 | 0 | [27] |  |
|  | | R: TCTTCCTTTTCCTTTCATCC | |  | |  |  |  |  |  |  |  |  |  |  |  |
| mEstTcCepec6 | | F: CAATCTCAAACGCTCAAAAC | | [CT]4 [TGA] 4 | | 194 | 1 | 0 | 0 | 0 | 1 | 0 | 1 | 0 | [27] |  |
|  | | R: TTGATCAGGGTTCTGTTGAC | |  | |  |  |  |  |  |  |  |  |  |  |  |
| mEstTcCepec9 | | F: CGTTCAATCCTTCTCAGTTG | | [CT]4[CT]4 | | 260 | 1 | 0 | 1 | 0 | 1 | 0 | 1 | 0 | [27] |  |
|  | | R: CCATGGAAATTGCAGATAAC | |  | |  |  |  |  |  |  |  |  |  |  | |

***S2 Table (continuation...). Characteristics of 20 EST-SSRs markers used on the present work. Shown for each primer pair are the previously published forward and reverse sequence, repeat type, allelic amplitude (bp), number of alleles (Na) and polymorphic information content (PIC) for species of Theobroma.***

| **Loci** | **Sequence (5'-3')** | | **Motif** | | **Allelic Amplitude (bp)** | | ***T. cacao***  **(17)** | | ***T. grandiflorum* (2)** | | ***T*. ssp**  **(5)** | | **Total (24)** | | **Reference** |
| --- | --- | --- | --- | --- | --- | --- | --- | --- | --- | --- | --- | --- | --- | --- | --- |
|  |  | |  | |  | | **Na** | **PIC** | **Na** | **PIC** | **Na** | **PIC** | **Na** | **PIC** |  |
| mEstTcCepec16-2 | | F: TAAACCTTCCATCTCCCATT | | [CA]4[GA]3 | | 210-212 | 1 | 0 | 1 | 0 | 2 | 0.26 | 2 | 0.26 | [15]* |
|  | | R: TCCATAGCTCGCTTGAATTA | |  | |  |  |  |  |  |  |  |  |  |  |
| mEstTcCepec49 | | F: AGGACGATGAAGAGGAAAG | | [AT]9 | | 210 | 1 | 0 | 1 | 0 | 1 | 0 | 1 | 0 | In this study* |
|  | | R:ATTAGACACACACACGCACA | |  | |  |  |  |  |  |  |  |  |  |  |
|  |  | |  | | **Average** | | **24** | **0.257** | **0.8** | **0.015** | **1.75** | **0.199** | **3** | **0.334** |  |

*The SSR were described in the corresponding paper but not submitted in the NCBI/EMBL databanks
